# Oral anticoagulation in high risk Takotsubo syndrome: when should it be considered and when not?

**DOI:** 10.1186/s12872-018-0930-1

**Published:** 2018-10-29

**Authors:** Francesco Santoro, Thomas Stiermaier, Francesca Guastafierro, Nicola Tarantino, Ingo Eitel, Natale Daniele Brunetti

**Affiliations:** 1 Department of Medical and Surgical Sciences, University of Foggia, Viale Pinto n.1, 71100 Foggia, Italy; 2 University Heart Center Lübeck, Medical Clinic II (Cardiology/Angiology/Intensive Care Medicine) and German Center for Cardiovascular Research (DZHK), Partner Site Hamburg/Kiel/Lübeck, Lübeck, Germany

**Keywords:** Oral anticoagulation, Left ventricular thrombi, Stroke, Prognosis, Follow-up, Broken heart syndrome, Apical ballooning, Takotsubo syndrome

## Abstract

Standard pharmacological therapy in Takotsubo syndrome (TTS) is still debated and there is a lack of prospective data. In their recent work in BMC Cardiovascular Disorders Abanador-Kamper et al. found that stroke in TTS has an event rate of 2.8% after 30 days and 4.2% after 12 months and they question which patients need oral anticoagulation. According to our clinical data, TTS patients with LV thrombi may be at high risk of stroke. These patients are characterized by apical ballooning pattern, high prevalence of ST-elevation and higher troponin I levels. We have recently proposed a therapeutic algorithm for oral anticoagulation in TTS. In case of apical ballooning pattern and increased admission levels of troponin-I (> 10 ng/mL), oral anticoagulation should be considered, while in case of midventricular/basal ballooning or apical ballooning associated with troponin-I levels < 10 ng/ml, oral anticoagulation should not be considered. A simple combination of echocardiographic parameters (apical ballooning pattern),ECG data (ST-elevation at admission and persistent after 72 h) and laboratory values (troponin serum levels) could be useful for an appropriate therapeutic management of oral anticoagulation in TTS.

We read with great interest the article from Abanador-Kamper et al. entitled “Temporarily increased stroke rate after Takotsubo syndrome: need for an anticoagulation?” [1]. In this study 72 patients with Takotsubo Syndrome (TTS) were enrolled and all were evaluated by cardiac magnetic resonance imaging during the acute phase and 2 months later. The stroke rate was 2.8% after 30 days and 4.2% after 12 months. Patients with stroke presented with apical ballooning and no one of them received prior anticoagulation.

Left ventricular (LV) thrombus formation was found in one patient (1.3%) with acute stroke. However, the real rate of LV thrombi may have been underestimated because thrombus formation can happen even 2 weeks after the acute event [2]. Stroke could also be a trigger for TTS due to the dysfunction of central autonomic network associated with cerebral infarction, especially involving the territory of middle cerebral artery or basilar artery [3]. Unfortunately, Abanador-Kamper et al. did not provide additional information regarding serum levels of troponin and ECG data.

In a multicenter study enrolling 541 TTS patients we found that 12 patients (2.2%) developed LV thrombi (all female presenting with apical ballooning pattern) [4]. Among these patients, 2 out of 12 (17%) had a stroke before anticoagulation initiation. These patients were characterized by a high prevalence of ST-elevation and higher troponin I levels. Troponin I levels > 10 ng/mL were the only predictor of LV thrombosis (normal values = 0.5 ng/ml).

According to this data we proposed a therapeutic algorithm for oral anticoagulation (Fig. 1). In case of an apical ballooning pattern and increased admission levels of troponin-I (> 10 ng/mL), oral anticoagulation should be considered, while in case of mid-ventricular/basal ballooning or apical ballooning associated with troponin-I levels < 10 ng/ml, oral anticoagulation should not be considered. Moreover, we also found that the presence of persistent ST-elevation during the first 72 h after admission is associated with LV thrombosis [5].

**Fig. 1:**
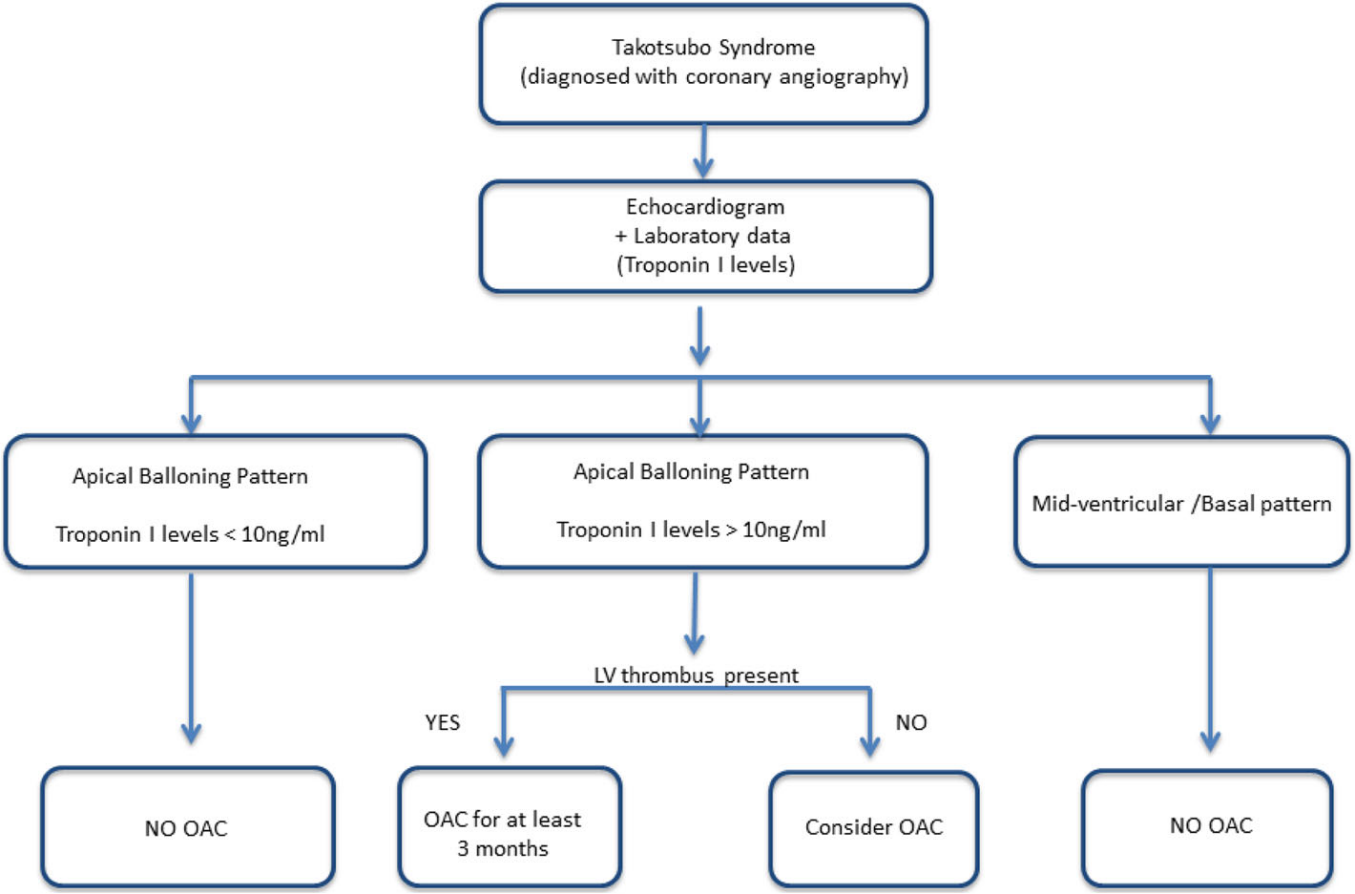
Therapeutic algorithm proposal for oral anticoagulation (OAC) management during the acute phase of Takotsubo syndrome. This Figure has been reproduced from Santoro et al. *Journal of the American Heart Association*, 2017;6: e006990

A simple combination of echocardiographic parameters (apical ballooning pattern), ECG data (ST-elevation at admission and persistence after 72 h) and laboratory values (troponin serum levels) could be useful for an appropriate therapeutic management of oral anticoagulation in TTS.

The high rates of stroke during the first 30 day after TTS remarks the urgent need of randomized trials assessing the role of anticoagulation in TTS.
