# Supplementary material for: Oral anticoagulation in high risk Takotsubo syndrome: when should it be considered and when not?
Source: BMC Cardiovasc Disord. 2018 Oct 29;18:205. doi: 10.1186/s12872-018-0930-1 (PMC6206855; doi:10.1186/s12872-018-0930-1)
Supplement: Related texts

# Reply: Response to “Oral anticoagulation in high risk Takotsubo syndrome: When should it be considered and when not?”

**Authors:** Nadine Abanador-Kamper, Lars Kamper, Judith Wolfertz, Marc Vorpahl, Patrick Haage, Melchior Seyfarth

**Affiliations:** 3 grid.490185.1 Department of Cardiology, Helios University Hospital Wuppertal, University Witten/Herdecke, Wuppertal, Germany; 4 0000 0000 9024 6397 grid.412581.b Center for Clinical Medicine Witten/Herdecke University Faculty of Health, Wuppertal, Germany; 5 grid.490185.1 Department of Diagnostic and Interventional Radiology, Helios University Hospital Wuppertal, University Witten/Herdecke, Wuppertal, Germany

We thank the authors for this important contribution and agree that prospective RCTs are urgently needed to define a therapeutic algorithm in patients with TTS and the need for anticoagulation.
